# Effects of pediatric first aid training on preschool teachers: a longitudinal cohort study in China

**DOI:** 10.1186/1471-2431-14-209

**Published:** 2014-08-24

**Authors:** Feng Li, Xiaoyang Sheng, Jinsong Zhang, Fan Jiang, Xiaoming Shen

**Affiliations:** 1 Department of Children and Adolescents Health Care, MOE-Shanghai Key Laboratory of Children’s Environmental Health, Xin Hua Hospital affiliated Shanghai Jiaotong University School of Medicine, 1665 Kongjiang Rd, Shanghai 200092, China; 2 Department of Developmental and Behavioral Pediatrics, Shanghai Pediatric Translational Research Institute, Shanghai Children’s Medical Center affiliated Shanghai Jiaotong University School of Medicine, MOE-Shanghai Key Laboratory of Children’s Environmental Health, 1678 Dongfang Rd, Shanghai 200127, China

**Keywords:** Preschool teachers, Knowledge retention, Pediatric first aid, Training

## Abstract

**Background:**

Unintentional injuries are a major cause of death among children. Data suggest that the retention of knowledge and skills about first aid declined over time. The purpose of this study was to assess the effects of pediatric first aid training among teachers.

**Methods:**

A stratified random sampling method was used to select 1,067 teachers. The selected trainees received pediatric first aid training. Follow-up assessments were conducted 6 months, 9 months and 4 years following the training. A standardized collection of demographics was performed, and participants were given a questionnaire to indicate knowledge of and emotions about first aid.

**Results:**

In the pretest, 1067 people responded with a mean of 21.0 correct answers to 37 questions, whereas in the post-test period, the mean score increased to 32.2 correct answers of 37 questions (*P* <0.001). There was a decrease in scores from post-test to 6 months, 9 months and 4 years after the training. However, the mean at the 6-month, 9-month and 4-year marks were higher than the pretest mean (*P* < 0.001). A total of 82.8% of the participants achieved a pass mark of 80% or above; 42.8% of participants achieved the pass mark at 6 months, 41.7% at 9 months and 11.7% at 4 years (compared with pre-test, *P* < 0.001). The mean score of the subjects’ emotions in the post-test period increased to 81 (*P* < 0.001). The mean scores of emotions at 9 months or 4 years were higher than the pretest mean (*P* < 0.001). At the 4-year mark, the majority of preschool staff (>70%) had administered correct first aid for injuries.

**Conclusions:**

This study demonstrated that the acquisition of knowledge, both short and long term, significantly improves. Despite appreciable decreases in knowledge long term, knowledge retention was modest but stable.

## Background

Childhood injury remains among the leading causes for childhood morbidity and mortality [1]. In the United States, injuries are the leading cause of death, disabilities, and health care utilization for children [2]. Injury alone accounts for almost one-half of all deaths in preschool-aged children in the USA [3]. Non-fatal injuries also cause a tremendous socioeconomic burden, as nearly one in four children is injured each year seriously enough to require medical attention, resulting in $17 billion dollars in medical costs [4]. The leading causes of nonfatal injuries for children ages 0 to 14 include falls, being struck by or against something, being cut or pierced, drowning, burns, and suffocation [5]. In China, injury accounts for one-third of all deaths in children aged 1 to 4 years and one-half of all deaths in children between 5 to 9 years of age [6]. Preschools are important locations in which to focus on the prevention of injuries and diseases in children because situations requiring first aid are often encountered there. The response time in emergency situations is critical, but the first aid provided must be performed properly to prevent further complications and to potentially save lives [7]. The correct first aid approach in childhood emergencies can be life-saving [8].

In schools, the person closest to the child and the first to apply first aid is often a preschool teacher. We have reported that the level of first-aid knowledge among preschool staffs in Shanghai was low [9]. Therefore, it is vital that preschool teachers be provided with first-aid knowledge and practical training [9], and teaching basic first aid should be compulsory in schools [10]. First aid training for regulated daycare providers may contribute to children’s health and safety in the daycare setting [11]. In 2005, the American Academy of Pediatrics (AAP) brought its national pediatric first aid course, pediatric first aid training for caregivers and teachers (PedFACTs). The PedFACTs course is designed to give caregivers and teachers the education and confidence that they need to care effectively for sick or injured children. In 2007, the program of PedFACTs in nurseries and kindergartens started in Shanghai of China. The program is aimed at equipping teachers with the appropriate first aid knowledge and skills to better care for the children. The purpose of this research was to evaluate the effectiveness of the PedFACTs in equipping teachers with appropriate first aid knowledge so that they can skillfully care for the children. While several studies have investigated the retention of knowledge and skills about first aid [12,13], a decline in resuscitation knowledge over time has been shown in many reports [14-16], and there is limited research evaluating these issues in PedFACTs. In addition to the assessment of first aid knowledge, evaluation of attitudes and behavior for first aid provision is also very important. However, there also have been few studies on preschool teachers’ emotions toward first aid situations. The long term knowledge level and emotions of first aid after PedFACTs has not been investigated. A further aim, therefore, was to establish to what extent knowledge levels and emotions are retained 6 months, 9 months and 4 years after the PedFACTs.

## Methods

This study was designed as a longitudinal study from 2008 through 2013. A pretest was conducted, followed by the intervention and an immediate post-test evaluation of learning outcomes in 2008. This process was followed by 6-month, 12-month and 4-year post-test evaluations to assess the retention of learning outcomes. A stratified random sampling method was first used to identify 1067 subjects in Shanghai, as previously described [9]. The selected teachers were trained in pediatric first-aid in a children’s hospital. The PedFACTs course focuses on what to do if a child in your care suddenly becomes ill or gets injured. The curriculum was derived from American PedFACTs courses and the American text book 《pediatric first aid for caregivers and teachers》; [17] was translated into Chinese and modified. All subjects had a 4-hour classroom course and all content is presented by a PedFACTs instructor. To ascertain the effectiveness of the PedFACTs, the participants’ knowledge was assessed at five stages:

1. Before the candidates received their PedFACTs.

2. Immediately upon completion of the PedFACTs.

3. Six months after the completion of the PedFACTs.

4. Nine months after the completion of the PedFACTs.

5. Four years after the completion of the PedFACTs.

At all stages, all participants in the study sat for an invigilated examination. The brief surveys assessed knowledge retention and emotions connected to first aid situations. The purpose of the invigilated exam was to ensure that access to course material was denied and that the test was completed in the half-hour time period. 1,067 participants completed the survey at stages 1 and 2. At stages 3, 4 and 5, three hundred subjects were independently drawn from the same 1,067 sampling frame using the statistical software package SPSS (version 17.0, SPSS Inc., Chicago, IL, USA) to participate in the examination 6 months, 9 months and 4 years after their first refresher in some kindergartens.

Before and after the training, a descriptive questionnaire was administered, which was divided into three sections as previously described [9]. Section A focused on demographic information of the participants. Section B was comprised of 37 simple-choice questions on the knowledge of the treatment of common children’s emergencies (Additional file 1). One point was awarded for each correct answer, total scores were computed as a sum of each item score (range 0–37). A score of 80% or greater was required to pass, in accordance with examination guidelines from the American Academy of Pediatrics. Section C addressed emotions toward first aid situations (Additional file 1). Emotions connected with first-aid situations were calculated on the basis of seven questions, and responses ranged from the most favorable alternative (100 points) to the least (0 points), which were taken from another first-aid training study [7]. The index measured negative emotions (afraid, anxious, stressed, passive, weak, puzzled, helpless) versus positive (safe, calm, relaxed, active, strong, engaged, confident). A high score is equivalent to a high degree of positive (low degree of negative) emotions. At stage 5, we asked subjects if they had ever witnessed childhood injuries in their work 4 years after the PedFACTs and how they dealt with the injury.

The study was approved by the Institutional Review Board and the Committee on Research Involving Human Subjects at Xinhua hospital, and the research was carried out in compliance with the Helsinki Declaration. Written informed consent was obtained from all participants. The research has adhered to strengthening the reporting of observational studies in epidemiology (STROBE) guidelines. The research design and methodology are presented in Table 1.

**Table 1:** Research design and methodology

**Section**	**Content**
Recruitment	
	1282 preschool staff were recruited from 1193 nurseries and kindergartens
Measurement	
	Baseline: 37-item survey, confidence and emotions toward first aid
	Post-intervention: 37-item survey, confidence
	6-month follow-up: 37-item survey, confidence and emotions toward first aid
	9-month follow-up: (see 6-month follow-up)
	4-year follow-up: (see 6-month follow-up), plus encountering
Intervention	
	4-hour course taught in classroom
	Course consists of lectures, video footage
	First aid Content:
	➢ Difficulty Breathing
	➢ Controlling Infection, Bleeding, and Swelling
	➢ Bone, Joint, and Muscle Injuries
	➢ Loss of Responsiveness, Fainting
	➢ Convulsions, Seizures and Head Injuries
	➢ Allergic Reactions, Bites and Stings
	➢ Poisoning and Burns
	➢ Eye Injuries and Oral Injuries
Retention	
	Baseline: 1067 preschool staff
	Immediate post-intervention: 1067 preschool staff
	6-month follow-up: 208 preschool staff
	9-month follow-up: 278 preschool staff
	4-year follow-up: 274 preschool staff

### Data analysis

All data were entered into SPSS 17.0 for Windows (version 17.0, SPSS Inc., Chicago, IL, USA) for statistical analysis. For non-continuous data we reported proportions and Chi square (χ^*2*^) test was used for comparison. For continuous data, an analysis of variance or Student’s t test was used to compare the scores based on groups. A level of P < 0.05 was considered statistically significant for all analyses.

## Results

### Knowledge

A total of 1,067 subjects participated in the training. In the post-test period (stage 2), the 1067 subjects were fully followed up. Of the 1,067 participants, 0.3% was male, and 99.7% were female. In sum, 62.0% were healthcare teachers, and 38.0% performed other jobs. A total of 30.8% of the group had previously taken a first-aid training course. Finally, at stages 3, two hundred and eight selected from the 1,067 participants (19.5%) were retested 6 months after the training; at stages 4, two hundred and seventy-eight selected from the 1,067 subjects (26.1%) participated in the examination 9 months after the training; and at stages 5, two hundred and seventy-four selected from the same 1,067 subjects (25.6%) come for retesting 4 years after the training. Ninety-two participants (at stage 3), twenty-two participants (at stage 4) and twenty-six participants (at stage 5) dropped out in later time points, because of lost contact. There was no statistically significant difference in demographic characteristics and pretest score among subjects in the five stages. In Table 2, the following variables are provided for all participants: district, age, occupation year, and staff categories in the five stages. Scores of knowledge in the five stages were significantly higher in preschool staffs who were healthcare providers, younger staff and those from a rural district. Comparisons of mean scores for all PedFACTs tests are presented in Table 2.

**Table 2:** **Comparison of the mean scores for all PedFACTs tests**^**a**^

**Variables**	**Pretest scores (mean ± SD)**	***P***	**Initial post-test scores (mean ± SD)**	***P***	**Post-test scores at month 6 (mean ± SD)**	***P***	**Post-test scores at month 9 (mean ± SD)**	***P***	**Post-test scores at year 4 (mean ± SD)**	***P***
District		<0.001		0.014		0.009		0.095		0.336
Urban	20.5 ± 4.5		31.99 ± 3.22		27.8 ± 4.2		27.7 ± 4.7		24.6 ± 4.4	
Rural	21.6 ± 4.6		32.46 ± 3.0		29.4 ± 4.5		28.7 ± 4.8		24.1 ± 4.3	
Age(years)		<0.001		<0.001		<0.001		<0.001		<0.001
≤30	22.0 ± 4.5		33.0 ± 2.4		29.5 ± 4.5		29.8 ± 4.9		25.5 ± 4.6	
31-40	21.3 ± 4.5		32.9 ± 2.7		29.3 ± 4.3		28.5 ± 4.4		25.5 ± 4.2	
≥41	20.0 ± 4.6		31.0 ± 3.6		27.0 ± 3.9		26.7 ± 4.6		23.2 ± 4.1	
Working years		<0.001		<0.001		0.005		<0.001		0.018
≤5	21.8 ± 4.3		32.70 ± 2.6		28.8 ± 5.0		28.7 ± 4.7		23.7 ± 4.2	
6-10	22.2 ± 4.6		32.94 ± 2.6		30.4 ± 4.0		30.6 ± 4.3		25.7 ± 4.4	
≥11	20.3 ± 4.6		31.8 ± 3.4		27.8 ± 4.0		27.4 ± 4.6		23.9 ± 4.3	
Staff categories		<0.001		0.177		0.463		0.012		0.045
Healthcare providers	21.5 ± 4.6		32.31 ± 3.1		28.6 ± 4.3		28.7 ± 4.8		24.7 ± 4.7	
Teachers	20.1 ± 4.5		32.04 ± 3.2		28.1 ± 4.4		27.2 ± 4.6		23.6 ± 3.8	

^**a**^The scores were calculated as the sum of the correct answers by the participants.

**Figure 1:**
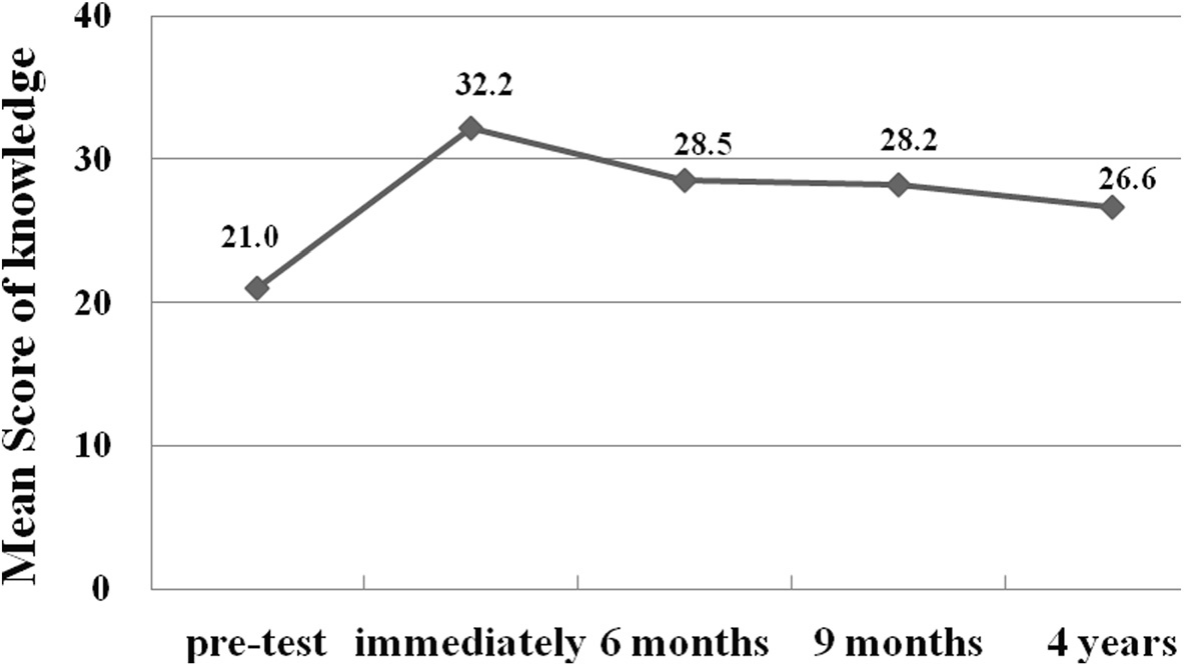
Scores for the written assessment of first aid knowledge at each stage in PedFACTs (maximum score is 37).

**Figure 2:**
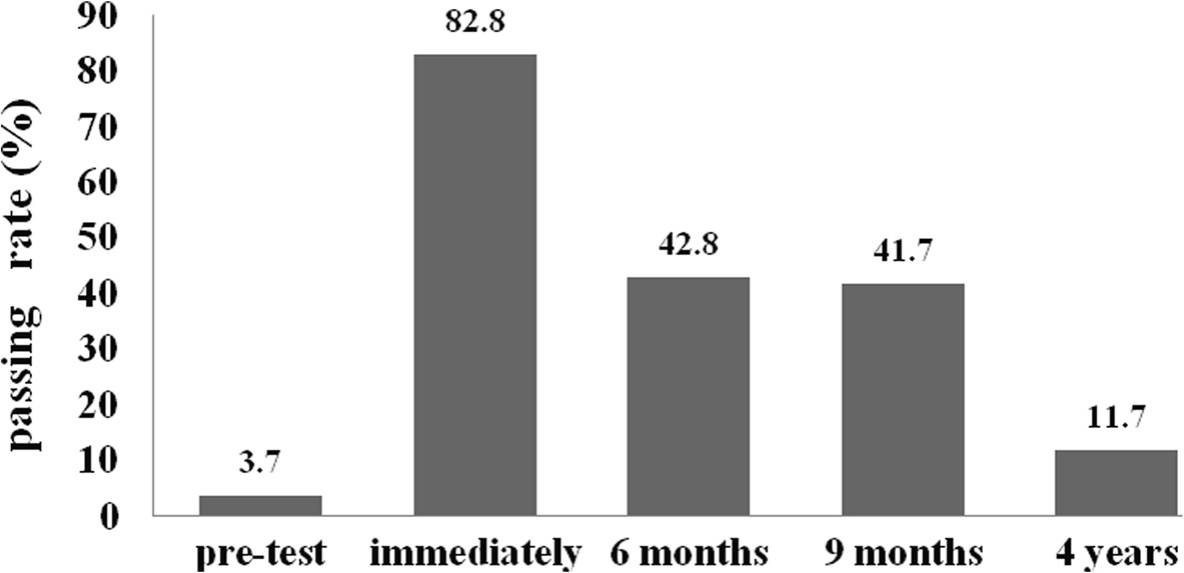
The passing rates at each stage in PedFACTs.

At stage 1, in the pretest period before training, 1,067 people responded with a mean accuracy of 21.0 correct answers out of 37 questions, whereas in the post-test period (stage 2), the mean scores increased to 32.2 (*P* < 0.001). At stage 3 (6 months after training), stage 4 (9 months after training) and stage 5 (4 years after training), the mean scores of students’ pediatric first aid knowledge were 28.5, 28.2 and 26.6 respectively. There was a statistically significant decrease in scores from post-test to 6 months, 9 months and 4 years after the training (*P* < 0.001). However, the mean at the 6-month, 9-month and 4-year marks were higher than the pretest mean (*P* < 0.001). Figure 1 illustrates the scores at the five stages in a curve of a marked increase following training to modest decay over time, stabilizing at a level significantly higher than the baseline.At stage 1, prior to the participants receiving the training, 3.7% achieved the pass mark of 80%. At stage 2, when the course examination was undertaken, 82.8% of the participants achieved a pass mark of 80% or above. At stage 3, 42.8% of participants achieved the pass mark of 80%; 41.7% passed at stage 4 and 11.7% at stage 5 (higher compared with pre-test, p < 0.001). Figure 2 illustrates the passing rates at each stage.

### Emotions

**Figure 3:**
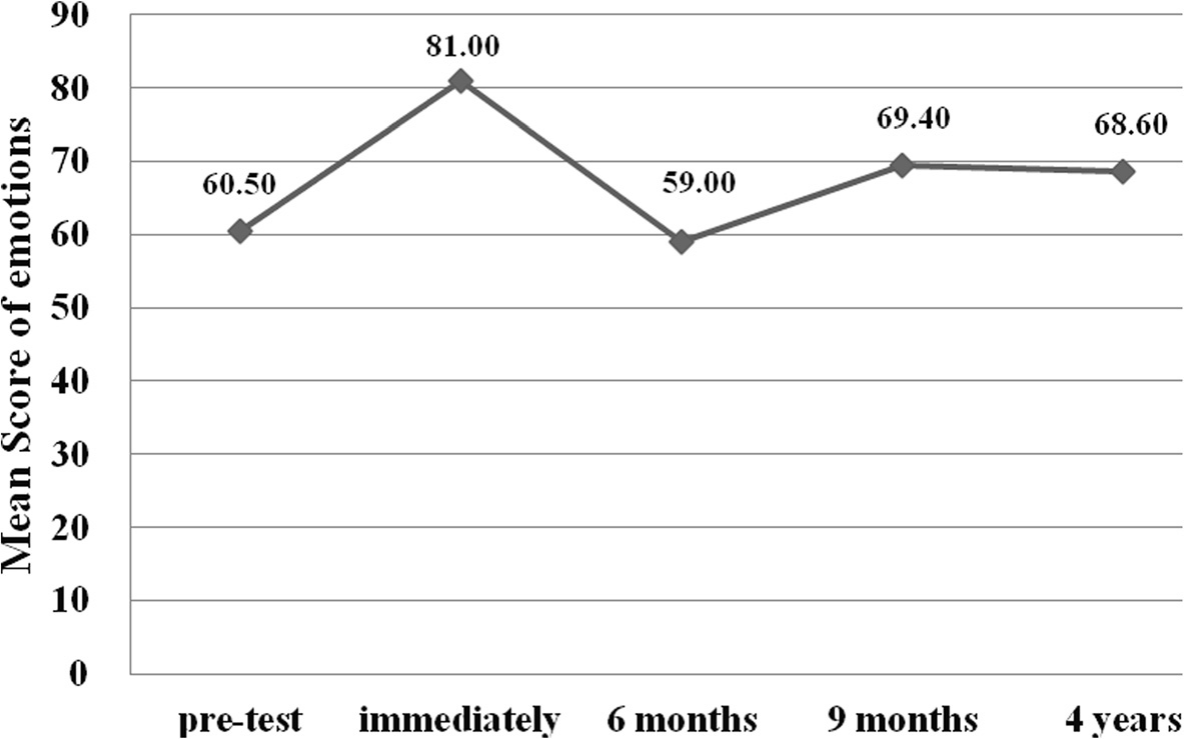
Mean scores of emotions connected to first aid situations at each stage in PedFACTs.

The mean score of subjects’ emotions connected to first aid situations in the pre-test was 60.5. The baseline score was not statistically different among the five stages (*P* > 0.05). Immediately following the training, the mean score of the subjects’ emotions in the post-test increased to 81 (*P* < 0.001). The decrease in scores of emotions from stage 2 to stage 3, 4 and 5 was found to be statistically significant (*P* < 0.001). Additionally, when the mean scores at stages 4 or 5 were compared with the scores at stage 1, the increase in scores was also statistically significant (P < 0.001). Figure 3 illustrates the mean scores of emotions connected to first aid situations at each stage in PedFACTs.

### Witnessed injuries and first aid

At the 4-year mark post-PedFACTs (stage 5), over half of the 274 respondents said that they had witnessed children with nosebleeds (90.9%), bleeding (60.6%) and swelling (58%). The respondents had witnessed injuries such as foreign object in eye (33.3%), bone injury (9.5%), asthma (6.6%), heatstroke (5.5%) and other injuries, as presented in Table 3. The majority of the preschool staff (>70%) who had witnessed injuries in children had administered correct first aid for nosebleeds, bleeding, swelling and so on. Correct responses regarding first-aid for choking, a coughing child (41.2%) and bites to the tongue (42.9%) were low (see Table 3).

**Table 3:** Percentages of staff who witnessed injuries and performed the correct first aid response to the injuries

**Injuries**	**Staff witnessed No. (%) (N = 274)**	**Correct first aid care**	**Correct response No. (%)**
Nosebleeds	249 (90.9)	pinch the soft parts of the nose and press against the bones	230(92.4)
Bleeding	166 (60.6)	place firm, direct pressure on the wound	120(72.3)
Swelling	159 (58.0)	apply cold compress, wrap, and elevate the injured body part	155(97.5)
Foreign object in eye	91(33.2)	pull the upper lid over the lower lid	82 (90.1)
Fainting	55 (20.1)	lay the child on her back and loosen her tight clothing	27 (49)
Convulsive seizures	37 (13.5)	position the child on his left side first	30 (81.1)
Burns	26 (9.5)	place the burned area in cool water	21(80.8)
Bone injury	26 (9.5)	rest and call EMS	23(88.5)
Bites to the tongue	21(7.7)	apply pressure with a piece of gauze or cloth to stop the bleeding	9(42.9)
Asthma	18 (6.6)	give children the asthma reliever medicine	13 (72.2)
Heatstroke	15(5.5)	cool the child immediately and call EMS	7 (46.7)
Insect stings, any stinger	14 (5.1)	move the child to a safe area and remove	11(78.6)
Punctures	10 (3.6)	soak the wound in clean water	7(70)
Choking and coughing child	7 (2.6)	do nothing except reassure the child and observe the child closely	2(28.6)
Swallowed poison	4(1.5)	remove traces of the poison from the child’s mouth first and then call EMS	4 (100)
Dog bites	4 (1.5)	care for the wound and check with animal control officer	3 (75)
Spinal injury	5(1.8)	avoid moving the child at all, and keep the neck and back aligned	5(100)

## Discussion

This is the first study to evaluate the impact of pediatric first aid training on caregivers and teachers (PedFACTs) and long term knowledge retention after PedFACTs. Our results showed excellent retention among teachers at 4 months, 12 months and 4 years post-training. Although knowledge and emotions tended to decline with time, improvements above the pretest were maintained at both 9 months and 4 years post-training. The results of this study demonstrate that the PedFACTs significantly improves knowledge levels and positive emotions toward first aid among preschool staff who attend the course. This correlates similarly with previous research investigating the effectiveness of the Advanced Trauma Life Support (ATLS) programme [12,18], the Advanced Trauma Nursing Course (ATNC) [13] and the American University of Armenia first aid training course for primary health care providers [15].

The learning that resulted from exposure to the course is likely a contributory factor to the improvement of outcomes. The principles of adult learning are purposively incorporated into the PedFACTs so that learning can be optimally effective. This study suggests that the acquisition of knowledge is a result of attendance in the PedFACTs. Only 39 participants prior to commencement of the course achieved the pass mark compared to an 82.8% pass rate immediately after the training. Following the training program, there was a significant acquisition in the preschool staff’s cognitive knowledge, and this study was also consistent with CPR training studies [19,20].

When scores on the long-term (6 months, 12 months and 4 years) changes were evaluated with the post-test, knowledge levels were found to decrease over time. However, despite the decline, the 28.2 mean score at the 9 months post-training mark was above the baseline score. Although the 4-year test showed a decreased mean of 26.6 correct answers, when compared with the pretest result (mean of 21.1 correct answers), it was higher by approximately 24%. As for the pass mark, 42.8% of participants achieved a pass rate six months after the course, 41.7% of participants achieved a pass rate nine months after the course, and 11.6% after four years, which were higher compared to 3.7% of participants prior to the training. This finding shows that the positive effect of education (although showing a trend of reduction) continues even after 4 years.

A large amount of literature in recent years has analyzed the efficacy of resuscitation training and has highlighted the fact that skills and knowledge decline over time [21]. The attrition of knowledge after Basic Life Support and Advanced Life Support courses has been well-documented [19,22]. Several studies have demonstrated limited retention of first aid knowledge and rapid deterioration of knowledge after initial training [16,23]. Madden reported that students had a significant deterioration in CPR cognitive knowledge 10 weeks following CPR training [24]. Tippett found significant deterioration in knowledge levels three months after completing the ATNC [13], and Ali et al. found significant deterioration in knowledge levels six months after completing the ATLS [25]. Most studies have also tested a combination of knowledge and skills, and skills are reported to decline faster than knowledge [26,27]. In our study, correct responses regarding first-aid for choking, a coughing child (41.2%) and bites to the tongue (42.9%) were also low. To maintain knowledge, refresher training is invariably required. Although recurrent training is recommended, the ideal time interval between refresher courses is not established in the literature. Research suggests that resuscitation training should be carried out at least every 3 to 6 months to prevent deterioration of skills and knowledge [21,28]. Another study suggests refresher training should be performed at least annually [14]. It is important, therefore, to evaluate the possible effects of teaching methods on knowledge retention and to explore new methodologies. Berden et al. [29] suggested that the acquisition and retention of CPR knowledge and skills is largely dependent on training and frequency of CPR instruction. Overtraining has been claimed to be of particular value in the retention of skills where the individual has no chance to ‘warm up’ [30], and previous reports on the positive effects of overtraining have also been published [30,31]. Wik et al. [32] reported that overtrained group, which had ten additional 3 minute training sessions shortly after the initial training, had significantly better skill retention than the control group. Turner et al. [33] suggested that paced testing using case-based tests after a life-support course can improve medical students’ retention of factual knowledge. Some studies have also reported that even simple retesting after 4 or 6 months improves retention several months later, as do short refresher courses [34-36].

Our study suggests that PedFACTs can improve staff members’ emotion levels. Scores on the emotions connected with first-aid situations remained significantly changed after the training and during the long-term follow-up period compared to baseline. One important barrier and a main concern of laypersons about administering first aid to acutely ill or injured people is the fear of making mistakes [37]. In our study, 4 years after PedFACTs, the majority of all respondents (N = 274) who had witnessed injured children had administered correct first aid for some injuries. In Austria, 68% of the participants of a study stated that they would not provide first aid because they feared doing something wrong [38]. Experts and instructors on first aid will agree that doing nothing is more dangerous than doing something that might be incorrect. Many people are afraid of providing first aid because they fear doing something wrong [38]. The results for emotions about first aid indicated that the preschool staff, at baseline, were prone to positive emotions (mean score was 60.5). Our study suggests that the intervention was effective in changing emotions about first aid after the training immediately. The score of emotions at 6 month after training was lower than that of baseline, but it is not statistically different. Its recovery to a more expected level at subsequent assessments (9 month and 4 years), this may be because that the teachers had witnessed some childhood injuries in the long term period and had experienced in dealing with the injuries, demonstrating a positive attitude toward emergencies. In the long-term follow-up period, preschool staff continued to express positive attitudes toward injured children and help reaction toward emergencies, and furthermore that they can assess the condition of the injuries and administer first aid for the injured children, this may be because that most of the teachers felt they were responsible for the management of the injuries in preschool and they believed they could improve the prognosis by early and correct intervention.

### Limitations

The acquisition of knowledge does not necessarily equate with improved care. First, within the design of our study, the influenced factors of knowledge retention between post-testing and re-testing were outside the control of the study. Second, we investigated the level of knowledge by a written questionnaire, and no practical skills could be tested in our setting. Third, because the same standardized tool was used at each data collection point, it is possible that participants could remember previous answers. Fourth, some teachers dropped out in later time points. We telephoned them or their kindergartens, but they did not respond to these efforts. But at stage 3, 4 and 5, most of the selected teachers (more than two thirds of selected three hundred teachers in each stage) participated in the examination at each stage. There was no any significant difference in demographic characteristics between those who come for the examination and those dropping out at each stage. Selection was random and selection bias may be small. Lastly, this local cross-sectional study cannot be generalized to our country. Further studies investigating the retention of knowledge immediately after the course and strategies employed to enhance the retention of knowledge should be welcomed so that these findings may influence the professional development of staff in child-care settings.

## Conclusions

This study demonstrated that the acquisition of knowledge in the short and long term significantly improves as a result of attending the PedFACTs. Our research findings suggest that despite appreciable decreases in knowledge in the long term, knowledge retention was modest, but stable, attitudes toward injured children remained positive. To retain knowledge levels after the completion of the PedFACTs, local initiatives aimed at improving retention of knowledge should be implemented. It is recommended that the provision of an ongoing structured program of PedFACTs is implemented.

## Abbreviations

PedFACTs: Pediatric first aid training for caregivers and teachers; CPR: Cardiopulmonary Resuscitation; SD: Standard Deviation.

## Competing interests

The authors have no conflicts of interest.

## Authors’ contributions

FJ and XMS conceptualized and designed the study, contributed to analysis, interpretation of data and approved the final manuscript as submitted. FL contributed to acquisition of data, carried out the initial analyses, drafted the manuscript, reviewed and revised the manuscript, and approved the final manuscript as submitted. XYS and JSZ were responsible for administrative, technical and material support. The authors revised the manuscript critically for important intellectual contents and approved the final manuscript as submitted.

## Pre-publication history

The pre-publication history for this paper can be accessed here:

http://www.biomedcentral.com/1471-2431/14/209/prepub

## Supplementary Material

Additional file 1 Knowledge and emotions on first aid questionnaire. Click here for file
