# Supplementary material for: Effects of pediatric first aid training on preschool teachers: a longitudinal cohort study in China
Source: BMC Pediatr. 2014 Aug 24;14:209. doi: 10.1186/1471-2431-14-209 (PMC4236654; doi:10.1186/1471-2431-14-209)
Supplement: Additional file 1 — Knowledge and emotions on first aid questionnaire. [file 1471-2431-14-209-S1.doc]

**Knowledge and emotions on first aid Questionnaire**

A number of questions on first aid knowledge and emotions will be asked, please choice the correct answer that you consider to be. Our survey is voluntary and anonymous, therefore you can set your mind at ease.

**B. First aid knowledge**

**1. When an emergency occurred in a preschool, what you should do first:**

a. Survey the scene first in the accidents

b. Call EMS

c. Call the parent or guardian

d. Provide first aid immediately

**2. Which of the following statements about first aid treatment is correct?**

a. You should rush into a burning building

b. Find out who is involved

c. You should care for crying child not the quiet child

d. If the scene is not safe, for the child who may have fallen, you should move the child to a safe place by using the cradle carry quickly

**3. A child fell and cut her knee on a sharp rock. To control the bleeding, you should:**

a. Place firm, direct pressure on the wound

b. Put a cold pack on the wound directly

c. Flushing the injured area with water

d. Cover the wound with an antibiotic ointment

**4. A child’s nose is bleeding. To control the nosebleed, you should:**

a. Have the child tilt her head back

b. Have the child lie down and place a cold pack on her nose

c. Ask the child to blow her nose until the bleeding stops

d. Pinch the soft parts of her nose and press against the bones of her face

**5. To reduce the risk of infection, clean small wounds by:**

a. Rubbing the dirt out with a soapy wash cloth

b. Pouring alcohol on the injured area

c. Flushing the injured area with running water

d. Wiping the wound with a commercially packaged antiseptic wipe

**6. A child twisted his ankle playing ball. To help reduce swelling, you should:**

a. Put ice directly on the child’s ankle

b. Place warm compresses on the ankle

c. Apply cold, wrap, and elevate the injured ankle

d. Place the injured part in a splint

**7. For splinters or other puncture wounds:**

a. Leave and splinter in place for parents to remove

b. Soak the wound in clean water

c. Apply an antiseptic to the wound

d. Use tweezers to remove them

**8. First aid care for fainting includes:**

a. Lay the child on his or her back, loosen any tight clothing and raise his legs

b. Lay the child flat on the floor

c. Lay the child down and using pillows to make her head higher than her feet

d. Have the child blow through a straw

**9. If you suspect that a child might have injured his neck or back, you should:**

a. Avoid moving the child at all, and keep the neck and back aligned as you find the child

b. Straighten out any awkward position so that the neck and back look straight

c. Encourage the child to stand up to see how badly injured he is

d. Sit the child up to take pressure off the brain

**10. To care for an obviously broken bone:**

a. Cover the injury with a hot pack and fix the injured area

b. Attempt to straighten any deformed area

c. Have the child assume a comfortable position and call EMS

d. Move the broken bone into the child’s body

**11. A child swallowed something that might be poisonous and is responsive. The first action you should take is:**

a. Give the child emetic

b. Remove traces of the poisonous from the child’s mouth first and then call EMS

c. Call the parent or guardian

d. Give the child plenty of fluid

**12. If a child is burned, the burn does not involve more than 1 percent of the body, and does not involve the full thickness of the skin, the person giving the first aid care should remove the child from whatever is causing the burn and then:**

a. Apply petroleum jelly to the burned area

b. Apply margarine to the burned area

c. Cover the burn with a dressing

d. Place the burned area in a container of cool water until the child no longer has pain

**13. An acceptable place to keep dangerous cleaning materials is:**

a. In tightly stoppered containers beneath the sink

b. In a well-labeled soft drink bottle with a tight stopper

c. In an inaccessible place (high, out of reach), and locked up

d. In a closed with the door tightly shut

**14. The first step in the first aid care for a child who has inhaled a poisonous substance is:**

a. Call the poison control center for advice

b. Identify the poison

c. Remove the child from the toxic area first

d. Have the child lie down

**15. A child is stung on his neck by a honeybee while having a picnic outside. The first thing you should do is:**

a. Honeybees leave a sack attached to their stinger in the skin, you should not get it out

b. Move the child to a safe area, remove any stinger by scraping it with a credit card and wash the area with soap

c. Don’t remove any body parts of the stinging insect and call EMS

d. Apply a hot compress over the skin

**16. A child was bitten by a stray dog that wandered into the playground. The dog is drooling and acting strangely and no one knows who owns the dog. Another caregiver takes the other children inside while you give first aid care to the injured child. The first thing you should do is:**

a. Apply antibiotic ointment over the bite area

b. Do not cover the wound so air can dry the wound

c. Thoroughly rinse wound with water and check with animal control officer

d. Let the child suck on small bit wounds and cover them with petroleum jelly

**17. A heavily dressed, very active child appears confused one summer day. Her skin is hot and dry. Her temperature is 106 °F. You should:**

a. Insist that she drink two glasses of a sports drink

b. Bring the child inside, cool her by pouring water on her skin and call EMS

c. Get someone to find a hose or small basin to cool her hands and feet

d. Pour a little water for her to drink slowly

**18. When blisters form in the area of the burn the person providing the first aid should:**

a. Wipe off a sewing needle with alcohol and puncture the blister so the fluid drains out

b. Press on the blister to see if the fluid will drain from it

c. Wash the blister with soap and water and then cover it with petroleum jelly

d. Cover the blister with a loose, protective dressing to try to keep the blister intact

**19. The first step in addressing an electrical burn is:**

a. Grab the child and pull them away from the source

b. Turn off the power source if possible and call EMS

c. Get some ice to apply to the burn

d. Call EMS

**20. A child has a foreign object in his eye. The first thing you should do is:**

a. Call EMS

b. Call the parent or guardian

c. Pull the upper lid over the lower lid

d. Cover the eye with a clean bandage

**21. A child gets a chemical in his eye. The first thing you should do is:**

a. Call the parent or guardian

b. Call the poison center

c. Call the child’s medical provider

d. Put on disposable gloves and flush the chemical from the eye with lukewarm wate

**22. The first thing in caring for a child with a penetrating injury to the eye is:**

a. Cover the eye with a paper cup

b. Gently remove the object

c. Call EMS

d. Flush the eye with running water

**23. When you begin recue breathing, which of the following statements is correct:**

a. For the child, you should seal your mouth around the child’s mouth, don’t pinch the child’s nose

b. For the infant, you tilt the head back and seal your mouth over the infant’s nose and mouth

c. Place all your hand on the center of the chest when you give the child chest compression

d. You should not loosen your hand pinching the child’s nose after you breathe in the child’s mouth

**24. A young child is crying after hitting his mouth on the edge of a table. He bites to his tongue, the first thing you should do is:**

a. Call EMS

b. Call the child’s parent or guardian to come get him

c. Apply pressure with a piece of gauze or cloth to stop the bleeding

d. Have the child lie down and use a cool cloth to wipe away the blood

**25. When a child is choking and coughing hard, you should:**

a. Begin rescue breathing

b. Clap your hand between the child’s shoulder blades

c. Give the child a drink of water

d. Do nothing, except reassure the child and observe the child closely

**26. Of the following emergencies, which is considered to be an immediate threat to life and require calling EMS?**

a. A child with a sprained ankle

b. A child bleeding from a wound to her mouth

c. A child choking

d. A child screaming

**27. When caring for a child who is having a convulsive seizure, you should:**

a. Force an object into the child’s mouth

b. Position the child on his left side and protect the child from injury

c. Keep the child flat on her back

d. Give the child water to drink

**28. A child has been stung by a bee. Within minutes her face and tongue are swelling and she is having difficulty breathing. These signs and symptoms are associated with:**

a. Seizures

b. Anaphylaxis

c. Asthma

d. Hypoglycemia

**29. When caring for a child with asthma, you should:**

a. Watch for signs of an asthma attack

b. Know where the child’s rescue medicine is kept

c. Know the proper techniques for giving the child’s rescue medicine

d. All of the above

**30. When caring for a child who is experiencing asthma and breathing hard, you should:**

a. Give the child water to drink and Lay the child flat on the floor

b. Give children the asthma reliever medicine

c. Give the child something to eat and comfort the child

d. Clap your hand between the child’s shoulder blades

**31. Before beginning CPR, determine:**

a. If there is any injury to the chest

b. That breathing, coughing and movement are absent

c. If the child has a medical condition

d. If the child has medical coverage

**32. When you are alone and caring for a choking child with a blocked airway, call EMS:**

a. Immediately

b. As soon as the object of obstruction is removed

c. After approximately 2 minute of performing first aid care

d. There is no need to call EMS

**33. To provide recue breathing:**

a. Tilt the head back

b. Pinch the nose of a child and breathe in the mouth

c. Seal your mouth over the infant’s nose and mouth

d. All of the above

**34. To perform chest compressions on an infant:**

a. Place the heel of one hand in about the center of chest

b. Compress the chest 1/2 to 2 inches deep

c. Compress slowly

d. Use two fingers to compress the child

**35. For rescue breathing, breaths should be delivered slowly, approximately once every:**

a. Five seconds b. Four seconds c. Three seconds d. Two seconds

**36. Start CPR for a child who is:**

a. Unresponsive or only gasping

b. Breathing normally

c. Responsive but not breathing normally

d. Having difficulty breathing and is responsive

**37. If a 9-month-old boy is choking and cannot cough, talk, or breathe, the first thing you should do is:**

a. Give back blows (slaps)

b. Put your finger in his mouth and do a finger sweep to try to find what is choking him

c. Give abdominal thrusts

d. Call EMS and wait for them to arrive

**C. Emotions toward first aid situations**

**If I came alone to a place where first-aid action was required, then I would feel:**

Afraid Safe

0 1 2 3 4 5 6 7 8 9 10

Anxious Calm

0 1 2 3 4 5 6 7 8 9 10

Stressed Relaxed

0 1 2 3 4 5 6 7 8 9 10

Active Passive

10 9 8 7 6 5 4 3 2 1 0

Strong Weak

10 9 8 7 6 5 4 3 2 1 0

Engaged Puzzled

10 9 8 7 6 5 4 3 2 1 0

Confident Helpless

10 9 8 7 6 5 4 3 2 1 0
